# Complete Genome Sequence of Marinobacter flavimaris LMG 23834^T^, Which Is Potentially Useful in Bioremediation

**DOI:** 10.1128/genomeA.00273-18

**Published:** 2018-04-19

**Authors:** Montserrat Palau, Nadia Boujida, Àngels Manresa, David Miñana-Galbis

**Affiliations:** a Secció de Microbiologia, Departament de Biologia, Sanitat i Medi Ambient, Facultat de Farmàcia i Ciències de l'Alimentació, Universitat de Barcelona, Barcelona, Catalonia, Spain; b Biotechnology and Applied Microbiology Research Group, Department of Biology, Faculty of Sciences, University Abdelmalek Essaâdi, Tetouan, Morocco

## Abstract

The complete genome sequence of the halophilic strain Marinobacter flavimaris LMG 23834^T^ is presented here. The genomic information of this type strain will be useful for taxonomic purposes and for its potential use in bioremediation studies.

## GENOME ANNOUNCEMENT

The halophilic Marinobacter flavimaris strain SW-145^T^ (= DSM 16070^T^ = KCTC 12185^T^ = LMG 23834^T^) was isolated from seawater at Daepo Beach (Yellow Sea) in South Korea, and it represents the type strain of Marinobacter flavimaris on the basis of phenotypic and phylogenetic data and genomic distinctiveness (1). We have sequenced the complete genome of Marinobacter flavimaris LMG 23834^T^ due to its usefulness from a taxonomic point of view and its potential use in bioremediation.

The description and delineation of novel taxa, with the inclusion of type strains as main representatives, should be based on as wide a data set as possible. In this context, approaches for incorporating genomics into microbial systematics are being developed and will become indispensable in the near future, with the sequencing of complete genomes of all type strains as the most critical challenge (2–4). In contrast, members of the genus Marinobacter, including Marinobacter flavimaris, are halophilic, hydrocarbonoclastic, and diazotrophic bacteria and are therefore especially useful for the bioremediation of hydrocarbon contaminants in both hypersaline environments and environments poor in nitrogenous compounds (5–7).

M. flavimaris LMG 23834^T^ was cultivated in marine agar (MA) and marine broth (MB), supplemented with 5% NaCl. Genomic DNA was isolated using a Real DNA extraction kit (Durviz S.L., València, Spain) according to the manufacturer’s specifications. DNA library preparation and sequencing (2 × 125 bp and 599.77× coverage), using an Illumina Hi-Seq platform, was performed by the Centre for Genomic Regulation (CRG, Barcelona, Catalonia, Spain). Assembly of the contigs was performed with the program a5-assembler (8); Prokka (9) was used for annotation of the genes, and Mauve (10) was used for genome sequence alignment and reordering of contigs according to the reference genome sequence of Marinobacter adhaerens HP15^T^ (NCBI reference sequence NC_017506). A total genome length of 4,463,800 bp was obtained from 65 contigs, with a contig *N*_50_ value of 394,355 and a GC content of 57.1%.

Genome annotation was also acquired from the NCBI Prokaryotic Genomes Annotation Pipeline (PGAP) (11), which revealed 4,154 genes, with 4,028 coding genes, 3 complete rRNAs (5S, 16S, and 23S), 50 tRNAs, and 5 noncoding RNAs (ncRNAs).

As M. adhaerens is the species most closely related to M. flavimaris (12), a comparison between the genome sequences of their respective type strains was conducted. This included digital DNA-DNA hybridization (dDDH) by the *in silico* genome-to-genome distance (GGD) method (13) and determination of the average nucleotide identity (ANI) by the OrthoANI algorithm (14). Values obtained for DDH (70.10%) and ANI (96.20%) were just above the thresholds for species delineation (70% and 95 to 96%, respectively) (4), suggesting that further analysis is needed to clarify the taxonomic status of M. adhaerens.

### Accession number(s).

This whole-genome shotgun project has been deposited in DDBJ/ENA/GenBank under the accession no. PSSW00000000. The version described in this paper is the first version, PSSW01000000.
